# Training and expertise in undertaking assisted vaginal delivery (AVD): a mixed methods systematic review of practitioners views and experiences

**DOI:** 10.1186/s12978-021-01146-3

**Published:** 2021-05-05

**Authors:** Claire Feeley, Nicola Crossland, Ana Pila Betran, Andrew Weeks, Soo Downe, Carol Kingdon

**Affiliations:** 1 School of Community Health and Midwifery, University of Central Lancashire, Preston, PR1 2HE UK; 2 Department of Reproductive Health and Research, World Health Organisation, 1211 Geneva 27, Switzerland; 3 Sanyu Research Unit, Liverpool Women’s Hospital Women and Children’s Health, University of Liverpool, Liverpool, L87SS UK

**Keywords:** Assisted vaginal birth, Assisted vaginal delivery, Competence, Training, Practitioners

## Abstract

**Background:**

During childbirth, complications may arise which necessitate an expedited delivery of the fetus. One option is instrumental assistance (forceps or a vacuum-cup), which, if used with skill and sensitivity, can improve maternal/neonatal outcomes. This review aimed to understand the core competencies and expertise required for skilled use in AVD in conjunction with reviewing potential barriers and facilitators to gaining competency and expertise, from the point of view of maternity care practitioners, funders and policy makers.

**Methods:**

A mixed methods systematic review was undertaken in five databases. Inclusion criteria were primary studies reporting views, opinions, perspectives and experiences of the target group in relation to the expertise, training, behaviours and competencies required for optimal AVD, barriers and facilitators to achieving practitioner competencies, and to the implementation of appropriate training. Quality appraisal was carried out on included studies. A mixed-methods convergent synthesis was carried out, and the findings were subjected to GRADE-CERQual assessment of confidence.

**Results:**

31 papers, reporting on 27 studies and published 1985–2020 were included. Studies included qualitative designs (3), mixed methods (3), and quantitative surveys (21). The majority (23) were from high-income countries, two from upper-middle income countries, one from a lower-income country: one survey included 111 low-middle countries. Confidence in the 10 statements of findings was mostly low, with one exception (moderate confidence). The review found that AVD competency comprises of inter-related skill sets including non-technical skills (e.g. behaviours), general clinical skills; and specific technical skills associated with particular instrument use. We found that practitioners needed and welcomed additional specific training, where a combination of teaching methods were used, to gain skills and confidence in this field. Clinical mentorship, and observing others confidently using the full range of instruments, was also required, and valued, to develop competency and expertise in AVD. However, concerns regarding poor outcomes and litigation were also raised.

**Conclusion:**

Access to specific AVD training, using a combination of teaching methods. Complements, but does not replace, close clinical mentorship from experts who are positive about AVD, and opportunities to practice emerging AVD skills with supportive supervision. Further research is required to ascertain effective modalities for wider training, education, and supportive supervision for optimal AVD use.

**Supplementary Information:**

The online version contains supplementary material available at 10.1186/s12978-021-01146-3.

## Plain language summary

During the late stages of childbirth, complications can occur which require rapid birth of the baby. This can be facilitated with instruments (usually forceps or a suction cup) or by surgery (caesarean section). In some circumstances, instrumental birth (also termed assisted vaginal delivery, AVD) may be a better option than caesarean section. AVD requires practitioners to develop skills, competence and expertise in the procedure. Our aim for this review was to examine practitioners’, funders’ and policy makers’ views about competence and expertise in AVD, how they can best gain this, the barriers and facilitators to implementing training packages, and their views, opinions and perspectives of their training. We included 27 studies (published 1985–2020), mostly from high-income countries. We had moderate confidence on one findings statement, with the rest assessed with low confidence. We found that practitioners valued extra training in AVD, observing others using the different instruments, and opportunities for clinical supervision, mentorship to gain experience, competence and expertise. We also found that, from the practitioners’ perspective, competence encompasses a number of inter-related skill sets; non-technical skills (e.g. effective communication with the labouring woman), broad clinical skills (e.g. capacity to assess the whole clinical picture) and technical instrumental skills (e.g. correct application of a vacuum cup to the fetal head, or capacity to turn the baby so it is in the right position). Practitioners also identified a number of barriers and facilitators that supported (or did not support) their training needs and development.

**Supplementary Information:**

The online version contains supplementary material available at 10.1186/s12978-021-01146-3.

## Background

Instrumental births mainly involve the use of forceps or a vacuum cup to expedite a birth, usually in situations of fetal compromise or for maternal benefit [1, 2]. Also known as ‘assisted vaginal delivery’ (AVD), it is a valuable tool during late second stage of labour where performing a caesarean section may not be possible, or safe, or acceptable to the woman [1, 2]. The prevalence of AVD use varies widely, both internationally and within countries [3]. However, as techniques of caesarean section have improved and become safer in the last few decades, in some contexts, the use of AVD has been substantially reduced in favour of caesarean section [1]. In some contexts low rates of AVD use occurs in parallel with overuse of caesarean sections, raising concerns about excess maternal morbidity or mortality due to unnecessary or unconsented surgery [1, 4]. In other contexts, low use of AVD and poor access to safe caesareans are associated with poorer neonatal outcomes, due to lack of access to safe and acceptable methods for managing maternal or fetal emergencies [1]. Forceps and vacuum births can both be undertaken safely, but concerns have been raised regarding inappropriate decision making about when to use them, and sub-standard levels of technical skills or training can cause iatrogenic harm. This could disincentivize their use in favor of a caesarean section (if this is option is possible) or even be a barrier to their use where they are the only technical solution available [1, 4, 5].

*Skilled* AVD use is recommended by the World Health Organization (WHO) as a safe alternative to caesarean section rates and as a means to improve maternal and neonatal outcomes when certain complications arise [2, 4]. Indeed, important improvements in fetal outcomes have been reported when optimal instrumental birth options are introduced into LMIC settings [1]. Our previous review [6] focused on women’s, partners’ and healthcare providers’ views and experiences of AVD. We found that views and experiences of AVD tended to fall somewhere between extremes [6]. Where indicated, AVD can be an effective, acceptable alternative to caesarean section. However, for childbearing women, the experience of AVD could be negatively impacted by the unexpected nature of events that precipitated their use. Particularly in high-income settings, this was associated with unmet expectations [6]. We also found that positive relationships, good communication, involvement in decision-making, and (believing in) the reason for intervention were important mediators of birth experience. Additionally, from the perspectives of professionals, attitudes and skills were simultaneously barriers (where substandard) and facilitators (where they were of high quality) for the acceptability of using AVD.

The aim of the current review was to improve the understanding of limitations, barriers and potential facilitating factors relating to expertise, training and competencies in AVD, from the perspective of maternity care practitioners, funders and policy makers.

The objectives of the review were to establish:

1. What expertise, training and competencies are required for optimal use of AVD?
2. What are the barriers and facilitators to achieving these levels of expertise and competence?
3. What are the barriers and facilitators to implementation of appropriate training?
4. What are practitioner views, opinions, perspectives and experiences on training for use of AVD?

## Methods

We used a systematic integrated mixed-methods design with the protocol published prior to commencing the review [7]. The review was carried out according to the protocol with the following exceptions: we were unable to undertake the planned meta-thematic synthesis due to the few qualitative studies found, and the low quantity and/or quality of the data presented in them. Due to this factor, we were also not able to carry out the pre-planned convergence matrix to assess the similarities/dissimilarities of findings between survey and qualitative data [7]. Therefore, we adopted a mixed-methods integrated review design, as per Noyes et al. [8]. This involved using both quantitative survey and qualitative data that was integrated to answer the research questions.

### Criteria for inclusion

Our focus was on the views, opinions, perspectives and experiences of maternity care providers, maternity funders and policy makers regarding the expertise, training and competencies required for optimal AVD. We also sought to examine the barriers and facilitators to achieving practitioner competencies and the implementation of appropriate training. We included primary studies that reported participants’ views, beliefs, concerns and experiences. These included studies using qualitative designs (e.g. ethnography, phenomenology) or qualitative methods for data collection (e.g. focus group interviews, individual interviews, observation, diaries, oral histories) and studies using quantitative designs such as surveys (e.g. questionnaires), or mixed methods approaches. There were no language restrictions. Searches were carried out from the inception data of each database, due to the potential value in examining historic use of instruments for expediting birth. Studies with a principle focus on breech presentation, multiple pregnancies, or where the lie of the fetus was transverse or oblique, or where participants were experiencing preterm birth, were excluded.

### Reflexivity

Transparent reflexivity throughout qualitative research is central to good practice [9]. Our interdisciplinary research team considered our potential biases prior to, and throughout the review to reduce potential impact upon the findings. CF and SD are midwives with extensive clinical experience, CK is a sociologist and all three have many years’ experience of maternity care research. All three believe that AVD can be a positive experience for women, if done well, with skill and with respect for women. However, all three recognise that it can be distressing and damaging if carried out without skill, respect and compassion. NC, is a health researcher who held prior beliefs about the importance of respectful care and communication for women throughout the maternity episode including AVD. ADW is an obstetrician who has practiced AVD in both the UK and Uganda over the last 25 years. He has seen perinatal deaths prevented by it, and debriefed women who have suffered trauma from it. He therefore sees both its potential benefits and harms, and believes that personal teaching and mentoring is critical to a good outcome. His personal preference is for routine ultrasound to determine fetal head position, non-rotational forceps and manual rotation when required. He uses vacuum only for ‘outlet procedures’. APB is a medical officer with 20 years of experience in maternal and perinatal health research and public health, who held prior beliefs about the importance of women having the right to evidence-based and respectful maternity care and that narrowing worldwide disparities is crucial, including disparities in the appropriate and respectful use of AVD for improved outcomes.

### Search strategy

A predesigned search strategy included pilot searching and information specialist input to ensure a robust approach. Systematic searches were carried out in March/April 2020 in MEDLINE, CINAHL, PyschInfo, EMBASE and Global Index Medicus (that included AJOL and LILACS databases). Searches were carried out using keywords (see Table 1) for the Population, Intervention and Outcomes adapted as necessary for the individual database i.e. using MeSH terms. An example search strategy is shown in Additional file 1: example search strategy. Additional searches were carried out, including reference checking of the included studies, cross-checking with Google Scholar and reference checking of key international guidelines (The International Federation of Gynecology and Obstetrics, The Royal College of Obstetricians and Gynaecologists, The American College of Obstetricians and Gynecologists, The Society of Obstetricians and Gynecologists of Canada, The Royal Australian and New Zealand College of Obstetricians and Gynaecologists).

**Table 1:** Search terms

Midwife or midwives or midwifery or nurse-midwife or obstetrician or doctor or physician or dr or ob or obstetrics or nurse-midwives or obstetric nurse or nurse or trainee or registrar or practitioner or personnel or resident or medical officer or medical or provider or worker or specialist or attendant or graduate or professional or intern
Assisted vaginal delivery or assisted vaginal birth or ventouse or vacuum or kiwi or extraction or vacuum assisted delivery or forceps delivery or instrumental delivery or instrumental birth
Skill* or knowledge or competence or train* or education or expertise or instruction

### Study selection

Records were collated into Covidence systematic review software [10] and duplicates removed. Each title and abstract was screened against the inclusion/exclusion criteria by the lead author (CF) and screened independently by one of three reviewers (CK, NC, APB). At full-text stage, each record was screened by the lead author (CF) and screened independently by one of two reviewers (NC, CK). Discrepancies were resolved by consensus. The final list of included studies agreed among the reviewers.

### Data extraction and quality assessment

Study characteristics of the included papers were collected on a data extraction form: author & date, title, resource setting, country, study design, setting, population, participants, methods. Quality assessment of quantitative surveys was carried out using a critical appraisal checklist [11]. Quality assessment of qualitative studies was carried out using criteria from Walsh & Downe [12]. Quality assessment of mixed methods studies was carried out using the Mixed Methods Appraisal Tool (MMAT) tool [13]. All studies were graded A-D by discussion between two reviewers (CF/NC), where A: No, or few flaws; the study credibility, transferability, dependability, and confirmability is high, and D: Significant flaws that are very likely to affect the credibility, transferability, dependability, and/or confirmability of the study [14]. No disagreements were noted, and no study was excluded on the basis of quality as per our protocol. A detailed exposition of the quality assessments can be found in Additional file 2: QA.

### Data synthesis

A data-based convergent synthesis [8] was carried out; whereby all of the included studies were analysed and synthesised using the same methods. This involved extracting the findings data from each paper in short relevant sections (as opposed to line-by-line coding, or statistical extraction) and tabulating. The data across the studies were then grouped as initial descriptive themes in relation to the research question (1–4). A second iteration refined these further to ensure the descriptions captured the data adequately and generated ‘Statements of Findings’. These were subjected to GRADE-CerQual assessments [15] which indicate the degree of confidence to be placed in each findings statements. Assessments included minor, moderate, or substantial concerns regarding four domains: 1. methodological limitations of included studies; 2. relevance of the included studies to the review question; 3. coherence of the review finding; and 4. adequacy of the data contributing to a review finding. Then, based on an overall assessment of these four domains, confidence in the evidence for each review finding was assessed as high, moderate, low or very low [15]. As per Noyes et al. [8], data transformation in this instance was ‘qualitised’ as in, the findings are presented narratively. A detailed exposition of the data extraction can be found in Additional file 3: data extraction.

### Results

**Fig. 1:**
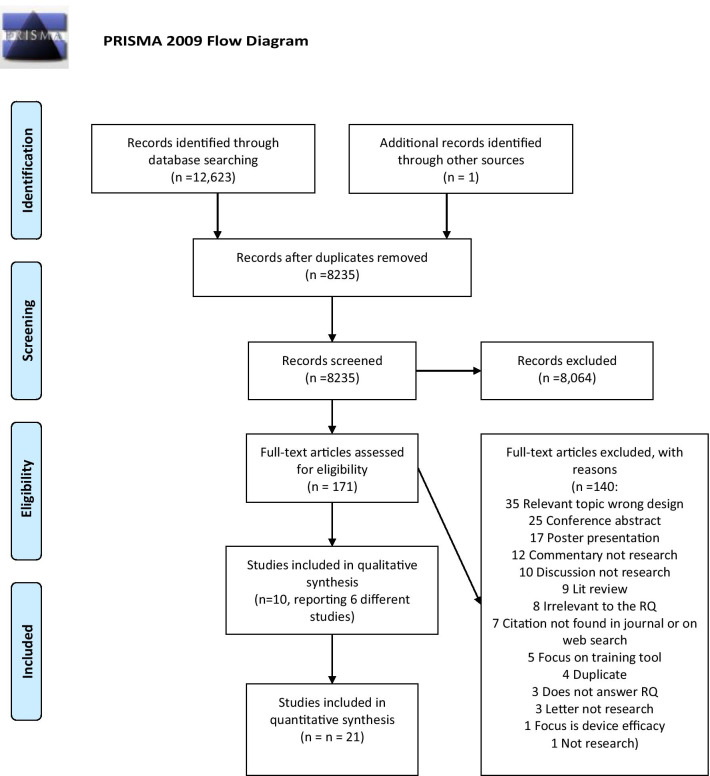
PRISMA 2009 Flow Diagram

From the searches, 12,623 hits were identified, and one further study was identified from an additional source. After 4389 duplicates were removed, 8064 records were discarded as irrelevant after reviewing title and abstract. Of 171 full-text papers screened, 140 records were excluded, with 31 papers [16–46] included into the review as shown in Fig. 1: PRISMA. No studies were found from the perspectives of funders or policy makers.

Of the 140 excluded records, 35 studies were on topic, but the wrong study design for example, pre/post-test training quasi-experimental studies. Details of all the excluded studies, and the reasons for exclusion can be found in Additional file 4: excluded studies.

Of note, the 31 included papers, several were from the same study (n = 4 [18–21], n = 2 [35, 43]), therefore, 27 *studies* were quality assessed, analysed and synthesised. Table 2 gives an overview of the characteristics and quality assessment of all included studies.

**Table 2:** Study characteristics

Author & date	Title	Resource setting	Country	Setting	Population	Participants	Design	Methods	QA grade
Al Wattar et al. 2017 [16]	Training on Kielland’s forceps: A survey of trainees’ opinions	HIC	England	Regional (not national)	Specialist trainee obstetricians	87/172 participated that were eligible	Quantitative	Cross-sectional survey	C
Alexander et al. 2001 [17]	An evaluation by focus group and survey of a course for Midwifery Ventouse Practitioners	HIC	England	National	Midwives who had completed ventouse advanced training	n = 8 focus group n = 18 survey (100% response rate)	Mixed methods	Focus group followed by survey	B
Bahl et al. 2008^a^ [18]	Qualitative analysis by interviews and video recordings to establish the components of a skilled low-cavity non-rotational vacuum delivery	HIC	UK	Two university teaching hospitals: Dundee & Bristol	Obstetricians and midwives	n = 10 obstetricians, n = 8 midwives	Qualitative	Interviews, vignettes, video recordings	A/B
Bahl et al. 2010^a^ [19]	Non-technical skills for obstetricians conducting forceps and vacuum deliveries: qualitative analysis by interviews and video recordings	HIC	UK	Two university teaching hospitals: Dundee & Bristol	Obstetricians and midwives	n = 10 obstetricians, n = 8 midwives	Qualitative	Interviews, vignettes, video recordings	A/B
Bahl et al. 2013^a^ [20]	Qualitative analysis by interviews and video recordings to establish the components of a skilled rotational forceps delivery	HIC	UK	Two university teaching hospitals: Dundee & Bristol	Obstetricians and midwives	n = 10 obstetricians, n = 8 midwives	Qualitative	Interviews, vignettes, video recordings	A/B
Bahl et al. 2013^a^ [21]	Decision-making in operative vaginal delivery: when to intervene, where to deliver and which instrument to use? Qualitative analysis of expert clinical practice	HIC	UK	Two university teaching hospitals: Dundee & Bristol	Obstetricians and midwives	n = 10 obstetricians, n = 8 midwives	Qualitative	Interviews, vignettes, video recordings	A/B
Biringer et al. 2019 [22]	Enhanced skills training in family medicine maternity care: Cross-sectional study of graduates’ experiences	HIC	Canada	National	Graduates of family medicine enhanced skills programs (2004–2014)	87/233 participated that were eligible	Quantitative	Cross-sectional questionnaire	C
Bofill et al. 1996a [23]	Forceps and vacuum delivery: A survey of North American residency programs	HIC	US	National	Residents (trainee O&G)	210/291 participated that were eligible	Quantitative	Purposive survey	C/D
Bofill et al. 1996b [24]	Operative Vaginal Delivery: A Survey of Fellows of ACOG	HIC	US	National	Fellow obstetricians	597/1600	Quantitative	Randomised survey	C/D
Chinnoock et al. 2009 [25]	An anonymous survey of registrar training in the use of Kjelland’s forceps in Australia	HIC	Australia	National	Registrar obstetricians (trainee)	197/303 participated that were eligible	Quantitative	Purposive survey	C/D
Crosby et al. 2017 [26]	An international assessment of trainee experience, confidence, and comfort in operative vaginal delivery	HIC	Ireland & Canada	International comparison	Trainee obstetricians	31/56 eligible Canadian trainees, 21/48 eligible Irish trainees	Quantitative	Purposive survey	C/D
Devjee 2015 [27]	A survey of health professionals on the current use of forceps / ventouse and skills training for operative vaginal delivery	UMIC	South Africa	Provincial	Healthcare workers (includes Interns /Medical Officers/ Community service officers /Registrars /Specialists)	197/250 (30 excluded for incompleteness) n = 15 Interns, n = 71 medical officers, n = 29 community service officers, n = 25 registrars, n = 15 specialists	Quantitative	Purposive survey	C/D
Eichelberger et al. 2015 [28]	Training needs in operative obstetrics for maternal–fetal medicine fellows	HIC	US	Regional (not national)	First year maternal–fetal medicine (MFM) fellows	86/100 participated that were eligible	Quantitative	Purposive survey	C
Evans et al. 2009 [29]	Where there is no obstetrician – increasing capacity for emergency obstetric care in rural India: An evaluation of a pilot program to train general doctors	LMI	India	Two sites Surat (Gujarat) and Jaipur (Rajasthan)	Medical officers	n = 17	Qualitative	Pilot study evaluation: program documents, facility observation, data abstraction at the facilities, and from semi-structured interviews with key informants (program and government staff, regional and international experts, trainees and trainers	B/C
Fauveau 2009 [30]	Is vacuum extraction still known, taught and practiced? A worldwide KAP survey	Low- middle	International study across Sub-Saharan Africa, Latin America and Caribbean Asia, the Pacific, Arab States, and Middle East Central Asia	International	Obstetricians, midwives, or public health experts specializing in maternal health	111/121 countries investigated	Quantitative	Rapid Knowledge—Attitude—Practice (KAP) survey	D
Friedman et al. 2020 [31]	Resident Attitudes Towards Caesarean Delivery in Canadian Obstetrics and Gynaecology Residency Programs	HIC	Canada	National	Residents (trainee O&G)	160/501 participated that were eligible	Quantitative	Cross-sectional purposive survey with open text option	B
Hamza et al. 2020 [32]	Vaginal operative delivery in Germany: a national survey about experience and self-reported competency	HIC	Germany	National	Residents (trainee), specialists and consultants	n = 653	Quantitative	Survey	B
Hankins et al. 1999 [33]	Forceps and vacuum delivery: Expectations of residency and fellowship training program directors	HIC	North America	National	Residents (trainee) and fellows	219/354	Quantitative	Purposive survey	C
Healyand Laufe 1985 [34]	Survey of obstetric forceps training in North America 1981	HIC	North America	National residency programs	Chairmen of programs	108/144 participated that were eligible	Quantitative	Purposive survey	C
Hodges et al. 2015^b^ [35]	Learning from Experience: Development of a Cognitive Task-List to Assess the Second Stage of Labour for Operative Delivery	HIC	Canada	Three large teaching hospitals in Toronto	Obstetricians identified as skilled	n = 20	Qualitative	Vignettes, video recordings, interviews, Delphi method	B/C
Powell et al. 2007 [36]	Vacuum and forceps training in residency: experience and self-reported competency	HIC	US	National	Chief residents (senior trainees)	n = 238 (20% of eligible participants) in first survey, n = 269 (23% of eligible participants) in second survey	Quantitative	Purposive survey repeated 1 year later due to low response rate in survey1	C
Ramphul et al. 2012 [37]	Strategies to enhance assessment of the fetal head position before instrumental delivery: a survey of obstetric practice in the United Kingdom and Ireland	HIC	UK & Ireland	International comparison	Obstetricians working in CLUs	323/423 participated that were eligible	Quantitative	Purposive survey with open text option	B
Robson and Pridmore. 1999 [38]	Have Kielland Forceps Reached Their ‘Use By’ Date?	HIC	Australia	One state (South Australia)	Obstetricians	23/29 participated that were eligible	Quantitative	Purposive survey	D
Rose et al. 2019 [39]	Forceps-assisted vaginal delivery: the landscape of obstetrics and gynecology resident training	HIC	US	National	Residents (trainees)	434/5061 participated that were eligible	Quantitative	Purposive survey	C
Sánchez del Hierro et al. 2014 [40]	Are recent graduates enough prepared to perform obstetric skills in their rural and compulsory year? A study from Ecuador	UMIC	Ecuador	Rural health centres in Southern Ecuador	Recently graduated medical doctors during their compulsory year of rural practice	90/92 participated that were eligible	Quantitative	Purposive survey	B
Sarangapani et al. 2018 [41]	Video-Based Teaching in Patient and Instrument Selection for Operative Vaginal Deliveries	HIC	Canada	One university site	Residents (trainees)	25/31 participated that were eligible-qualitative arm	MM overall, but qual data only to be used	Focus groups following training session	B
Saunier et al. 2015 [42]	French residents' training in instrumental deliveries: A national survey	HIC	France	National	Residents (1st years were excluded)	263/758 participated that were eligible	Quantitative	Purposive survey	C/D
Simpson et al. 2015^b^ [43]	Learning From Experience: Development of a Cognitive Task List to Perform a Safe and Successful Non-Rotational Forceps Delivery	HIC	Canada	Three large teaching hospitals in Toronto	Obstetricians identified as skilled	n = 17	Qualitative	Vignettes, video recording analysis	B/C
Smith 1991 [44]	GP trainees' views on hospital obstetric vocational training	HIC	UK	National	General practitioner trainees	765/1019 participated that were eligible	Quantitative	Randomised survey	C/D
Tang et al. 2012 [45]	Impact of introducing consultant resident on-call in a District General Hospital	HIC	England	One hospital site	Consultants, junior trainees and midwives	n = 17 (unclear how many of each professional group)	Mixed methods	Retrospective study and interviews	C/D
Wilson and Casson 1990 [46]	Babes in the Woods: Teaching the Use of the Vacuum Extractor	HIC	Canada	One hospital site	Family physicians	n = 23	Quantitative	Retrospective audit and survey (survey data to be used)	C

^a^Same study different research questions reported in the separate publications

^b^Same study different research questions reported in the separate publications

The 27 studies included n = 3 qualitative [18–21, 29, 35, 43], n = 3 mixed methods [17, 41, 45], n = 21 quantitative survey designs [15, 19, 21–25, 27, 29–33, 35–39, 41, 43, 45]. The majority were undertaken in high-income countries (n = 23), two were from upper-middle income countries [27, 40], one was from a lower-income county [29] and one survey included 111 low-middle countries [30]. The earliest study was 1985 [34] and the most recent was published in 2020 [32]. The studies included participants that were: trainee obstetricians [16, 23, 25, 26, 31, 33, 34, 36, 39, 42, 47], recent obstetric graduates [40, 41], obstetricians [22, 24, 32, 35, 37, 38, 43], GP/family medicine doctors [44, 46], midwives [17], medical officers [29], and multi-professional participants [18–21, 27, 45]. The quality of the included papers was generally poor whereby the majority of the survey studies scored C or C/D.

The convergent synthesis generated the descriptive themes and Statements of Findings (SoFs) -the development is shown in Table 2. Table 3 shows the summary of review findings and associated CERQual assessments (Table 4).

**Table 3:** Descriptive theme iteration and SoF development

Q	Early descriptive theme	Emergent themes	SoF	Studies contributing
Q1	Clinical skills to avoid instrumental birth	Non-technical skills	Non-technical skill expertise is essential to the optimal use of AVD	Bahl 2010; Bahl 2013b; Simpson 2015; Alexander 2001; Sarangapani 2018 [17, 19, 21, 41, 43]
	Behaviours			
	Decision making (multiple points of decision-making)			
	Clinical skills-assessing the clinical picture	Clinical skills-assessing the clinical picture	Broader clinical skills are essential to the optimal use of AVD	Bahl 2013b; Hodges 2015; Alexander 2001; Ramphul 2012; Sarangapani 2018; Simpson 2015 [16, 20, 34, 36, 40, 42]
	Additional tools	Technical skills	Technical skills expertise is essential to optimal use of AVD	Bahl 2008; Bahl 2013a; Simpson 2015 [17, 19, 42]
	Rotating the fetus			
	Angles and force			
	Checklist			
Q2	Specific training	Proactive teaching, specific training and supervision	Proactive teaching, specific training and supervision are essential to achieving competence and expertise	Evans 2009; Smith 1991; Simpson 2015; Hamz 2020; Hankins 1999; Healy and Laufe 1985 [28, 31–33, 42, 43]
	Close supervision			
	Proactive teaching			
	Poor communication by staff (rationale/lack of explanation)			
	Implementing learning following training			
	Exposure to being taught AVD	Exposure and gaining experience	Exposure to AVD including a range of instruments and the provision of opportunities to gain experience is essential to achieving competency and expertise	Bofill 1996a; Bofill 1996b; Healy and Laufe 1985; Saunier 2015; Crosby 2017; Eichelberger 2015; Powell 2007; Rose 2019; Sanchez del Heirro 2014; Smith 1991; Wilson and Casson 1990; Alexendar 2001; Al Watter 2017; Chinnock 2009; Fauveau 2009; Friedman 2020; Hamza 2020; Robson and Pridmore 1999; Sarangapani 2018 [15, 16, 22–25, 29–31, 33, 35, 37–41, 43, 45, 46]
	Experience			
	Expectation of the course/program leads			
	Lack of exposure/opportunity			
	Safety and/or litigation concerns	Attitudes /beliefs	The attitudes and beliefs evident in the training programme, work environment or individual practitioners appears to influence the attainment of competence and continued use of AVD	Bofill 1996a; Bofill 1996b; Hankins 1999; Devjee 2015; Eichelberger 2015; Powell 2007; Ramphul 2012; Robson and Pridmore 1999; Smith 1991; Wilson and Carson 1990 [23, 24, 27, 33, 36–38, 44, 46, 47]
	Attitudes /beliefs			
Q3	Challenges when teaching others	Access (or lack of) to teachers/training	Access (or lack of) to training courses and/or willing clinical mentors influences the implementation of AVD training	Devjee 2015; Powell 2007; Healy and Laufe 1985; Rose 2019; Wilson and Casson 1990; Sarangapani 2018; Bahl 2008; Al Watter 2017 [15, 17, 26, 33, 35, 38, 40, 45]
	Lack of opportunity			
	Lack of experienced teachers/mentors			
	Increased consultant cover			
	Access to teachers/ training			
	Colleagues proactive in teaching skills of AVD			
Q4	Wanting more training	Training needs	Access to training in AVD is sought after and valued by some practitioners	Biringer 2019; Al Watter 2017; Devjee 2015; Bofill 1996b; Wilson and Casson 1990; Powell 2007 [15, 21, 23, 26, 35, 45]
	Concerns regarding lack of training/skills			
	Training increases competence and confidence	Training relates to competence, confidence, job satisfaction and influences later clinical practice	Training enhances competence, confidence, job satisfaction and influences later clinical practice for some practitioners	Evans 2009; Alexander 2001; Biringer 2019; Eichelberger 2015; Powell 2007; Al Watter 2017; Chinnock 2009; Smith 1991 [15, 16, 21, 24, 28, 35, 43, 46]
	Increased job satisfaction			
	Competence influences later clinical practice			
	Videos as a supportive teaching tool	Supportive teaching tools	Practical teaching tools were valued by participants	Sarangapani 2018; Al Watter 2017; Devjee 2015; Healy and Laufe 1985; Rose 2019 [15, 26, 33, 38, 40]
	Simulation as a supportive teaching tool

**Table 4:** CERQual assessments

Q1	SoF	Studies	CerQual assessment	Explanation of assessment
1	**Non-technical skill expertise is essential to the optimal use of AVD**. These skills include behavioural skills i.e. demonstrating capability through confidence, situational awareness, teamwork, communication, good relationships with the women and professional behaviour. Decision-making skills are also essential e.g. the appropriateness of AVD, if so, where to carry out the AVD (room or theatre), which instrument to use and when to abandon AVD	5 studies: Bahl 2010; Bahl 2013b; Simpson 2015; Alexander 2001; Sarangapani 2018 [17, 19, 21, 41, 43]	Low confidence	Due to low number of studies reporting on this finding
2	**Broader clinical skills are essential to the optimal use of AVD.** Clinical skills should encompass strategies that avoid AVD to optimise the opportunity for an SVD. Where AVD is clinically necessary, clinical skills include history taking, maternal and fetal observation information and abdominal palpation to include assessment of fetal descent into the pelvis. Skilled vaginal examinations that assessed fetal position, fetal station, fetal flexion, caput, moulding and engagement are essential. A minority suggest the skilled use of ultrasound to determine the fetal attributes prior to AVD Clinical skills should also include ensuring adequate analgesia for the women prior to AVD and the opportunity to debrief following the AVD	6 studies Bahl 2013b; Hodges 2015; Alexander 2001; Ramphul 2012; Sarangapani 2018; Simpson 2015 [16, 20, 34, 36, 40, 42]	Low confidence	Methodological concerns and limited data
3	**Technical skills expertise is essential to optimal use of AVD.** Technical skills expertise should encompass proficiency in rotating the fetus (manually or with forceps); appropriate timing of applying the instrument (between contractions); competence in the application of specific instruments; aptitude in the use of angle and consideration of the necessity of an episiotomy	3 studies Bahl 2008; Bahl 2013a; Simpson 2015 [17, 19, 42]	Low confidence	Due to low number of studies reporting on this finding

Q2	SoF	Studies		
4	**Proactive teaching, specific training and supervision are essential to achieving competence and expertise.** Structured training and/or proactive teaching and supervision facilitates competence. Active tuition and close supervision throughout AVD by experienced colleagues skilled in teaching is particularly beneficial. This is influenced by the program (i.e. doctors training) expectations of learning AVD, thus providing the opportunities to achieve competency. Lack of teaching and/or specific training is a barrier to achieving competence in AVD	6 studies Evans 2009; Smith 1991; Simpson 2015; Hamza 2020; Hankins 1999; Healy and Laufe 1985 [28, 31–33, 42, 43]	Low confidence	Methodological concerns and limited data
5	**Exposure to AVD including a range of instruments and the provision of opportunities to gain experience is essential to achieving competency and expertise.** Competency development is facilitated by exposure and support with different instruments for AVD and different techniques required for varying clinical situations i.e. rotational forceps. Access to repeated opportunities to perform AVD was a key facilitator to developing the skill set to achieve competence. Conversely, a lack of exposure to gain experience, particularly in relation to forceps was a barrier to achieving competence and confidence	19 studies Bofill 1996a; Bofill 1996b; Healy and Laufe 1985; Rose 2019; Saunier 2015; Crosby 2017; Eichelberger 2015; Powell 2007; Sanchez del Heirro 2014; Smith 1991; Wilson and Casson 1990; Alexendar 2001; Al Watter 2017; Chinnock 2009; Fauveau 2009; Friedman 2020; Hamza 2020; Robson and Pridmore 1999; Sarangapani 2018 [15, 16, 22–25, 29–31, 33, 35, 37–41, 43, 45, 46]	Moderate confidence	Methodological concerns are mitigated by the number of studies that generated the SoF
6	T**he attitudes and beliefs evident in the training programme, work environment or individual practitioners appears to influence the attainment of competence and continued use of AVD.** Preferences towards or against specific types of instruments appears to influence practitioner use which may in turn, influence attaining competence in and or all AVD options. In some situations, fears regarding litigation or through a lack of support staff influenced decision-making towards caesarean section over AVD	10 studies Bofill 1996a; Bofill 1996b; Hankins 1999; Devjee 2015; Eichelberger 2015; Powell 2007; Ramphul 2012, Robson and Pridmore 1999; Smith 1991; Wilson and Carson 1990 [23, 24, 27, 33, 36–38, 44, 46, 47]	Low confidence	Substantial methodological concerns regarding the survey studies

Q3	SoF	Studies		
7	**Access (or lack of) to training courses and/or willing clinical mentors influences the implementation of AVD training.** Enabling facilitative environments include appropriate staffing usually by more experienced obstetricians who are skilled to teach AVD. This may be negatively influenced by clinical mentor’s preferences toward a particular instrument (forceps or vacuum), whereby trainees may not develop skills across all instrumental options. Training may also be impeded by a lack of teaching skills whereby articulating procedures and decision-making can be challenging	8 studies Devjee 2015; Powell 2007; Healy and Laufe 1985; Rose 2019; Wilson and Casson 1990; Sarangapani 2018; Bahl 2008; Al Watter 2017 [15, 17, 26, 33, 35, 38, 40, 45]	Low confidence	Methodological, adequacy concerns limit the confidence assessment

Q4	SoF	Studies		
8	**Access to training in AVD is sought after and valued by some practitioners.** Some practitioners seek further and/or advanced training to develop their AVD skills citing the need to gain more experience and seek more supervision. Others (obstetric trainees, GPs on rotation and obstetricians) specifically reported additional training with forceps was necessary	6 studies Biringer 2019; Al Watter 2017; Devjee 2015; Bofill 1996b; Wilson and Casson 1990; Powell 2007 [15, 21, 23, 26, 35, 45]	Low confidence	Methodological, adequacy concerns limit the confidence assessment
9	**Training enhances competence, confidence, job satisfaction and influences later clinical practice for some practitioners.** For practitioners who were not obstetricians (medical officers, midwives) who were upskilled to manage emergency obstetrics, training enhanced feelings of competence in clinical and technical AVD skills. Furthermore, the training enhanced their confidence and increased their job satisfaction. For trainee doctors, not yet in a specialty, extra training including AVD influenced their decision to practise obstetrics. For obstetric trainees, access to specific AVD training enhanced their confidence and competence in AVD, particularly in the use of forceps	8 studies Evans 2009, Alexander 2001; Biringer 2019; Eichelberger 2015; Powell 2007; Al Watter 2017; Chinnock 2009; Smith 1991 [15, 16, 21, 24, 28, 35, 43, 46]	Low confidence	Methodological, coherence and adequacy concerns limit the confidence assessment
10	**Practical teaching tools were valued by participants.** Specifically designed training using a range of tools was reported to enhance participants learning. Videos and simulation training were viewed positively as formalised education to complement clinical learning	5 studies Sarangapani 2018; Al Watter 2017; Devjee 2015; Healy and Laufe 1985; Rose 2019 [15, 26, 33, 38, 40]	Low confidence	Methodological, adequacy concerns limit the confidence assessment

Bold values are the titles of the findings

### What expertise, training and competencies are required for optimal use of AVD?

Three SoF’s were relevant to this question (all with low confidence), they related to non-technical, broader clinical and technical AVD skills.

#### Expertise in non-technical skills

Five studies [17, 19, 21, 41, 43] highlighted non-technical skills that included behaviours associated with demonstrating capability through confidence, situational awareness, teamwork, communication, good relationships with the women, professional behaviour and decision-making. Bahl et al. [19] identified three essential behavioural elements; calmness, confidence/assertiveness and self-awareness, including self-knowledge of professional limitations [19]. The importance of decision-making skills were highlighted across the five studies [17, 19, 21, 41, 43]. While these are also essential for clinical practice in general, the studies emphasised the importance of key decision-making points for AVD specifically, including clinical skills that can be used to avoid instrumental birth [17, 19, 41]. For example, a midwife who had been trained to use the ventouse commented:

‘…the art…is in diagnosing the babies you don’t attempt to do a ventouse on. OPs, OTs, brows, etc., high heads–diagnosis during VEs in labour p.167.’ [17]

Where AVD was deemed appropriate, other decisions were identified i.e. where to carry out the AVD (labour room or operating theatre) [21, 41, 43], which instrument to use [21] and when to abandon AVD in favour of caesarean section [19, 21, 43]. Such decision-making involved the weighing up of multiple and simultaneous factors. For example, the likelihood of success in a particular case influenced the decision of whether to perform the AVD in the labour room, or to recommend transfer to an operating theatre before attempting the procedure [21].

#### Clinical skills

Six studies [17, 21, 35, 37, 41, 43] reported on the broader clinical skills/assessment of the clinical picture required for optimal use of AVD. In the first instance, Bahl [21] captured specific clinical practices (as distinct from the decision-making skills noted above) from obstetricians and midwives deemed as experts, of techniques they used to encourage a spontaneous vaginal birth in a situation where there was a chance that this might be achieved safely, but with a view to moving rapidly to instrumental birth if necessary.

‘If she has been pushing for 1 h and the vertex is visible, one option is to continue pushing for another period of time to see if she can have a spontaneous vaginal delivery…….If the head was crowning but held back by the perineum, it will be an option to offer episiotomy…..Check if she has passed urine or whether it would be appropriate to catheterise and empty the bladder to allow delivery……If there is no concern regarding CTG and the contractions are inadequate, consider putting up syntocinon to augment the contractions p.337.’ D5 [21]

Where other techniques had been tried, or where AVD was clearly the safest option, other clinical skills were highlighted across the six studies [17, 21, 35, 37, 41, 43]. These included assessing the woman’s history and current clinical picture and ensuring fetal/maternal signs of wellbeing before proceeding to AVD rather than an emergency surgical birth [17, 21, 35]. Sarangapani et al. [41] emphasised their belief in the importance of abdominal palpation skills which they divided into three components:

‘(1) palpating the abdomen for a clinical assessment of fetal size; (2) palpating a central gap, typically a handbreadth between the xiphoid process and the fetal buttock, as an indication of good head descent into the pelvis; and (3) performing a bimanual examination, with one hand on the abdomen measuring the amount of fetal head (in fingerbreadths) above the pubic symphysis as an indication of fetal head descent p. 1165.’ [41]

Additionally, practitioners’ beliefs in the importance of skilled vaginal examinations was highlighted across the six studies [17, 21, 35, 37, 41, 43]. One study provided a detailed analysis relating to vaginal examinations: Ramphul et al. [37] surveyed 323 obstetricians (trainees and consultants) and found that between 98 and 99% of the surveyed obstetricians identified the following as benefits of undertaking skilled vaginal examinations; establishing fetal position, fetal station, the degree of cephalic caput and moulding of the fetal skull; and the degree of engagement of the presenting fetal part. While the survey found proportional differences between consultants and trainees, respondents of all grades agreed with the importance of assessing asynclitism of the presenting fetal part, flexion of the head, and estimated fetal size [37]. However, some obstetricians (18.7%) and trainees (23.1%) used ultrasound to aid diagnosis of the fetal head position if they had difficulty in determining fetal position digitally [37]. Simpson et al. [43] provided criteria regarding the vaginal examination to guide AVD decision-making i.e. the cervix must be fully dilated; membranes must be ruptured; and the presenting part must be at least at the level of the ischial spines [43].

Only three papers (two studies) referred to the importance of ensuring women had adequate analgesia [21, 35, 43] prior to AVD, arguably an essential clinical skill. One recommended offering *‘a debrief with parents, both at the time of delivery and the following day (*id*eally) p. 591′* [43]. No studies mentioned the need to discuss the procedure with the woman, and to obtain authentic consent from her prior to proceeding.

#### Technical skills and expertise

The technical skills required for optimal AVD were highlighted in three papers, two of which were based on the same dataset [18, 20, 43]. The studies noted the need for specific skills where the presentation was not directly occipito-anterior, including expertise in the positioning of the forceps blades for rotational (Kielland) forceps [18], and in manual rotation followed by the use of forceps for direct traction (non-rotational forceps delivery) [43]. More generally, these skills included the need for specific technical expertise in judging the timing of applying the instrument (between contractions); competence in the application of specific instruments; aptitude in the appropriate angle of traction; and consideration of the necessity, or not, of an episiotomy [18, 20, 43].

### What are the barriers and facilitators to achieving these levels of expertise and competence?

Three SoF’s were relevant to this question; proactive teaching, specific training and supervision (low confidence), exposure to opportunities to gain experience in AVD (moderate confidence), the attitudes and beliefs of mentors, training programs and/or localised clinical placements (low confidence).

#### Proactive teaching, and specific training and supervision

Six studies [29, 32–34, 43, 44] highlighted facilitatory factors. These were: proactive teaching, specific training, and targeted supervision to enable the development of competence in AVD. For example, structured and specific training provided to medical officers for emergency obstetric care in rural India was reported as highly beneficial [29], particularly for skills in undertaking AVD where the capacity to perform safe caesarean sections was limited. In a high income country (UK); GP trainees survey respondents [44] reported that the more teaching they had received the more relevant they thought obstetric training (including the performance of AVD) was to the provision of obstetric care in the community. In a national survey administered to all obstetricians in Germany [32], those actively tutored by their colleagues were significantly less likely to report incompetence when using forceps or vacuum extraction highlighting the benefits of proactive teaching and specific training.

Two surveys [38, 41] found barriers to gaining competencies in AVD. Sarangapani et al. [41] carried out a mixed-methods study with trainee obstetricians and found several challenges to their learning, including poor communication by mentors:

‘…[I]…find teaching on this topic tends to happen on call and it is kind of rushed. They don’t go through a super detailed rationale because there isn’t always time and some people have developed a gestalt of doing things, and they can’t always elucidate why they are doing it p. 1166.’ [41]

Additional barriers related to lack of opportunities. Preferences of existing staff, and the norms of particular hospitals, negatively influenced clinical learning opportunities (e.g. *‘instrument selection is largely institutionally dependent*’ [41]). Lack of theoretical or formalised education was also noted [41]. This was echoed by another survey [38] of 23 trainee obstetricians, where virtually all respondents expressed concern about a lack of training opportunities with Kielland forceps.

A survey of obstetric residency program directors [33] inferred that program expectations influenced the opportunities available to the trainees to develop competency in AVD, both negatively and positively:

‘All programs expected their graduates to be proficient in outlet deliveries… 97% of residency and 100% of fellowship directors expected proficiency; for vacuum, 92% expected proficiency in both types of training programs. In contrast, only 38% of residency and 48% of fellowship program directors expected proficiency with mid [cavity]-forceps, while 69 and 73% expected proficiency with mid-vacuum p.26.’ [33]

#### Exposure to AVD including a range of instruments and the provision of opportunities to gain experience

19 studies illustrated facilitators and/or barriers related to the degree to which trainees had exposure to AVD within their clinical practice areas, and, therefore, opportunities to gain experience [16, 17, 23–26, 30–32, 34, 36, 38–42, 44, 46, 47]. Issues related to exposure (or lack of) was particularly highlighted regarding the rate at which specific instruments were used locally [16, 17, 23–26, 30–32, 34, 36, 38–42, 44, 46, 47]. Inter-regional differences were found in several surveys [23, 24, 26, 34, 36, 39, 40, 42, 46] highlighting that the localised context influences the use and teaching of instruments. For example,

‘There was however a significant difference in number of [forceps assisted vaginal deliveries] FAVDs performed by region (p < 0.0001). Residents from the Midwest completed the most FAVDs compared to any other region, with 9% having completed > 30 FAVDs and 27% having completed 11–30 FAVDs p. 2.’ [39]

#### The attitudes and beliefs influence the attainment of competence and continued use of AVD

Some studies reported the cessation of teaching mid-pelvic operative vaginal deliveries citing safety and litigation concerns [23, 33]. Such fears were reflected in another study whereby some trainees reported they would not use any forceps, at all, in their clinical practice [38] also due to fears of safety and/or litigation. This concern was echoed by experienced obstetricians in a survey carried out in South Africa [27], who reported that only twenty-one (10%) of the participants continued to do operative vaginal deliveries (OVD) (approximately 1–2 every 3–6 months) following their training. The authors hypothesised that the majority of survey participants would resort to caesarean section because of the fear of litigation and the lack of skilled personnel in attendance in OVD [27].

Conversely, related to the vacuum extractor, a survey in Canada [46] showed how exposure to the successful use of instrumental birth can change attitudes and beliefs over time:

'If you administered your vacuum extractor questionnaire to me now I would give much more enthusiastic responses about the use of the vacuum. The midwives here do the normal deliveries, and the doctors are called for complications. We only have a small hand-held vacuum extractor but it has seen me through many difficult deliveries p. 1722.' [trainee in Canada, now working in Ethiopia] [46]

### What are the barriers and facilitators to implementation of appropriate training?

One SoF was relevant to the research question; access (or lack of) to training courses and/or willing clinical mentors influences the implementation of AVD training (low confidence).

#### Access (or lack of) to training courses and/or willing clinical mentors influences the implementation of AVD training

Eight studies [16, 18, 27, 34, 36, 39, 41, 46] generated insights regarding the barriers/facilitators to the implementation of appropriate AVD training. In South Africa, a survey found of 197 participants, 84% had learned AVD from ‘essential steps in the management of obstetrics emergencies’ (ESMOE) training modules indicating good uptake when training was available [27]. Other studies reported that willing clinical mentors who were proactive in their teaching of AVD positively influenced appropriate training [16, 18, 34, 36, 39, 41, 46]. Conversely, two studies illustrated the negative influence of poor teaching, as a result of which the articulation of AVD skills was reported as challenging and some participants reported conflicting messages between mentors [18, 41]. In another survey of 81 trainees, (35%) were not even sure if training on the use of AVD was provided in their units, or not [16].

### What are the views, opinions, perspectives and experiences of maternity care practitioners on training for use of AVD?

The final three review findings suggested that access to training sought after and is valued (low confidence); it enhances competence, confidence, job satisfaction and influences later clinical practice for some practitioners (low confidence); practical teaching tools were value (low confidence).

#### Access to good quality training in AVD is sought after and valued by some practitioners

Six studies [16, 22, 24, 27, 36, 46] found that participants sought further and/or advanced training to further develop their AVD skills or to gain more experience. For example, a survey in Canada [22] with family physicians who underwent an advanced fellowship, reported 69% of 87 respondents stated that they took the fellowship because they wanted more experience, and the same proportion did not feel ready to practise obstetrics (in general that included AVD) without supervision. In another survey [16], of 87 obstetric trainees, *‘the majority felt that they needed training in using Kielland's forceps (71/87, 81.6%)*’. These participants identified further training needs despite being on an obstetric trainee programme.

#### Good quality training enhances competence, confidence, job satisfaction and influences later clinical practice for some practitioners

Eight studies [16, 17, 22, 25, 29, 36, 44, 47] identified positive experiences of training. For practitioners who were not obstetricians (medical officers, midwives) who were trained to manage emergency obstetrics, training enhanced feelings of competence and confidence in clinical and technical AVD skills. Furthermore, the training enhanced participants’ confidence and increased their job satisfaction. A mixed methods study of midwives who had undertaken ventouse training [17] found 15/18 participants felt that becoming a midwifery ventouse-practitioner (MVP) had positively affected their overall midwifery practice, and enhanced confidence in abdominal palpation and vaginal examinations as well their ability to ‘handle’ a prolonged labour. One respondent said [17]– *‘I am much more likely to try everything to obtain a normal delivery… (Midwife 14 p. 168).’*

Likewise, family physicians who embarked on an advanced fellowship were much more likely to intend to practice obstetrics following the training [22]:

‘Eighty-two percent of participants indicated that the ability to access extra training influenced their decision to practise obstetrics. They cited that the fellowship made them more confident and more comfortable with intrapartum care. When asked if the fellowship was useful to their current practice, 75% rated it a 6 or 7 (on a 7-point scale where 7 was “essential”; mean rating 5.9); 93% said that they would choose to complete the extra training again p.534.’ [22]

#### Practical teaching tools were valued by participants

Five studies reported the value of practical and specifically designed training that incorporated different teaching modalities [16, 27, 34, 39, 41]. Videos and simulation training were viewed positively as an adjunct to clinical learning [27, 34, 41]. However, the authors from a study that introduced video based learning [41] emphasised that alternate teaching methods that *‘act as complementary tools to clinical teaching, but there was agreement that they should not replace hands-on clinical teaching (p. 1167).’*

## Discussion

The aim of this review was to improve understanding of the characteristics of competency and expertise in AVD, and to generate insights regarding the barriers and potential facilitating factors relating to expertise, training, implementation of and competencies in AVD. We identified 27 studies of health professionals’ views of training and competencies for AVD, mostly in high income countries.

We found that AVD competency comprises three inter-related components; non-technical skills, broader obstetric clinical skills and specific technical skills associated with particular instrument use. Practitioners felt that they needed additional training and exposure to AVD in practice to gain skills and confidence in this field, even after they had officially qualified (including those who had undertaken specialist obstetric training). Teaching aids such as simulation and video teaching tools were viewed as a valuable complement to clinical mentorship. However, the attitudes and behaviours of their colleagues and mentors, and the norms of their training and employing institution, were critical in determining if an individual practitioner would consider using AVD in routine practice, or not. This included the degree to which the fear of litigation in the employing organisation, and/or among peers and senior staff, influenced rapid recourse to caesarean section in preference to any kind of vaginal birth in three studies [23, 27, 33]. Good quality clinical mentorship or supervision was also particularly influential for trainees to gain competence, confidence and expertise in all instrumental births, or conversely was a negatively influencing factor.

Only three relevant qualitative studies were identified in our extensive searches. The majority of studies (23 out of 27 were from high-income countries). These limitations are reflected in our CERQual assessments, which were low for all except one summary of findings statement. This review also includes some older studies that may not be relevant for current practice. For instance, some of the studies refer to UK general practitioners undertaking AVD, but this cadre of doctors no longer routinely attends births in the UK. An important strength of the review is that it has located all studies in all languages that have been published to date, and it includes survey data. Our searches also found 28 studies that were either pre/post-test AVD training intervention quasi-experimental, RCT training intervention studies, or cross-sectional studies that examined maternal/neonatal outcomes following training [47–73]. Those studies did not meet our criteria for inclusion, but they do warrant further investigation in future reviews to determine the attributes, effectiveness and efficacy of good training that meet the needs of practitioners, and of service users.

Findings from our previous review [6] highlighted for both parents, the distress of unexpected interventions associated with AVD (including episiotomy, need for unplanned pain relief, such as epidural analgesia, and concern for possible iatrogenic harm to the baby of using instruments) may be mitigated by how health professionals communicate, both at the time of decision-making, and during the process. In the present review only three papers (two studies) referred to ensuring women had adequate analgesia and one discussed offering parents a debrief following AVD [18, 34, 42]. Although this knowledge may be implicit or assumed information, The Royal College of Obstetricians and Gynaecologists (RCOG) [48], the Royal Australian and New Zealand College of Obstetricians and Gynaecologists (RANZCOG) [49], and the Society of Obstetricians and Gynaecologists of Canada (SOGC) [50] guidelines for instrumental vaginal birth all highlight the importance of adequate postnatal care and counselling.

The 2020 update of the American College of Obstetricians and Gynaecologists (ACOG) [51] guidance on operative vaginal birth also include lists of prerequisites for AVD. Several of the studies we included in our review did generate checklists based on their research findings to establish the components: of a skilled low-cavity non-rotational vacuum delivery [17], of a skilled rotational forceps delivery [19], non-technical skills, cognitive or decision-making aids for instrumental births [18, 20, 34, 42]. These tools do not appear to have been formally evaluated in practice. Preparing clinicians and maintaining the proficiency in the recognition and management of events that are relatively rare such as emergency obstetrics is a challenge, including AVD and caesarean section [52–56]. Evidence on the effectiveness of the multiple methods in use (e.g. classes, simulation-based training methods, rehearsals, drills, interactive training) is suboptimal. Although, multidisciplinary training has been recognized as essential including clinical and non-clinical skills [57], identification of the most useful combination of methods remains uncertain and warrants future studies to rigorously assess effectiveness. This is particularly important in low- and middle-income countries where healthcare providers cannot rely on strong health systems, referral structure or appropriate supplies flow. AVD is one of the seven basic services as prerequisites for emergency obstetric care as defined by the WHO. The main reasons cited for the low rates in LMICs are known to include lack of skilled healthcare workers and lack of resources with the establishment of training programs for all skilled birth attendants a priority [58].

AVD is not without risks. In this review fear of bad outcomes and litigation were concerns reported by some clinicians. These factors coupled with suboptimal training and thus low levels of skills underpin the deprioritization of AVD in favour of caesarean section [58]. Low- and middle-income countries are most affected by this trend and the use of AVD is almost non-existent in these settings [3]. However, caesarean section is not without risks either and it is precisely in these countries and in resource constrained conditions where appropriate and skilled use of AVD could have a more significant impact by optimizing the use of caesarean section and improving perinatal outcomes [1]. Effective training programmes need to be revitalized in order to foster feasible alternative solutions for emergency obstetric care. This also includes close supervision by experienced consultants, as highlighted in a recent study [59]. In addition, research is on-going on new devices such as the Odon device, with a potentially safer profile and easier to use for all cadres of birth attendants that could possibly expand the options to expedite birth and improve maternal and perinatal outcomes when certain complications arise during labour [60–62].

## Strengths and limitations

The strengths of this study are that a comprehensive and rigorous search strategy was undertaken, including reviewing papers in other languages. While an evidence synthesis is an interpretative process, the risk of over or under interpretation of the data was minimized through author reflexivity to ensure that personal beliefs and values did not obscure important data within the included studies, and through rigor in study selection and analysis. However, there are some limitations; we were unable to undertake the planned meta-thematic synthesis due to the few qualitative studies found, and the low quantity and/or quality of the data presented in them. Due to this factor, we were also not able to carry out the pre-planned convergence.

## Conclusion

Most AVD techniques can be used safely in very low resource environments, by single practitioners. Most women prefer to give birth vaginally. Skilled, respectful AVD can be a safe, acceptable, and low-cost solution when birth needs to be expediated. Fear of bad outcomes and litigation are factors likely to be compounded when professional training is inadequate to ensure confidence and competence in clinicians’ skills in this area, or when local colleagues and seniors, or organisational norms, deprioritise AVD in favour of caesarean section. Our findings suggest that both pre- and post-registration training for maternity care practitioners needs to include the development of positive attitudes. Access to specific AVD training, using a combination of teaching methods, complements, but does not replace, close clinical mentorship from experts who are positive about AVD, and opportunities to practice emerging AVD skills with supportive supervision. Further research is required to ascertain effective modalities for wider training, education, and supportive supervision for optimal AVD use.

## Supplementary Information

**Additional file 1: Table S1.** Example search-MEDLINE.

**Additional file 2.** QA.

**Additional file 3.** Data extraction.

**Additional file 4.** Excluded studies.

## Data Availability

The search strategy, screening and data extraction tables have been supplied as Additional files within this submission.

## Abbreviations

AVD: Assisted vaginal delivery
