# Supplementary material for: Training and expertise in undertaking assisted vaginal delivery (AVD): a mixed methods systematic review of practitioners views and experiences
Source: Reprod Health. 2021 May 5;18:92. doi: 10.1186/s12978-021-01146-3 (PMC8097768; doi:10.1186/s12978-021-01146-3)
Supplement: Supplementary file 1 — Additional file 1: Table S1. Example search-MEDLINE. [file 12978_2021_1146_MOESM1_ESM.docx]

Supplementary File Table 1 Example search- MEDLINE

| **#** | **Query** | **Limiters/Expanders** | **Last Run Via** | **Results** |
| --- | --- | --- | --- | --- |
| S12 | S3 AND S7 AND S11 | Expanders - Apply equivalent subjects | Interface - EBSCOhost Research Databases | 4,069 |
|  |  | Search modes - Boolean/Phrase | Search Screen - Advanced Search |  |
|  |  |  | Database - MEDLINE with Full Text |  |
| S11 | S8 OR S9 OR S10 | Expanders - Apply equivalent subjects | Interface - EBSCOhost Research Databases | 1,827,948 |
|  |  | Search modes - Boolean/Phrase | Search Screen - Advanced Search |  |
|  |  |  | Database - MEDLINE with Full Text |  |
| S10 | (MH "Education, Professional+") | Expanders - Apply equivalent subjects | Interface - EBSCOhost Research Databases | 294,843 |
|  |  | Search modes - Boolean/Phrase | Search Screen - Advanced Search |  |
|  |  |  | Database - MEDLINE with Full Text |  |
| S9 | TI ( skill* or knowledge or competence or train* or education or expertise or instruction ) OR AB ( skill* or knowledge or competence or train* or education or expertise or instruction ) | Expanders - Apply equivalent subjects | Interface - EBSCOhost Research Databases | 1,648,418 |
|  |  | Search modes - Boolean/Phrase | Search Screen - Advanced Search |  |
|  |  |  | Database - MEDLINE with Full Text |  |
| S8 | (MH "Clinical Competence") OR (MH "Professional Competence+") | Expanders - Apply equivalent subjects | Interface - EBSCOhost Research Databases | 115,077 |
|  |  | Search modes - Boolean/Phrase | Search Screen - Advanced Search |  |
|  |  |  | Database - MEDLINE with Full Text |  |
| S7 | S4 OR S5 OR S6 | Expanders - Apply equivalent subjects | Interface - EBSCOhost Research Databases | 272,314 |
|  |  | Search modes - Boolean/Phrase | Search Screen - Advanced Search |  |
|  |  |  | Database - MEDLINE with Full Text |  |
| S6 | TI ( assisted vaginal delivery or assisted vaginal birth or ventouse or vacuum or kiwi or extraction or vacuum assisted delivery or forceps delivery or instrumental delivery or instrumental birth) ) OR AB ( assisted vaginal delivery or assisted vaginal birth or ventouse or vacuum or kiwi or extraction or vacuum assisted delivery or forceps delivery or instrumental delivery or instrumental birth ) | Expanders - Apply equivalent subjects | Interface - EBSCOhost Research Databases | 270,267 |
|  |  | Search modes - Boolean/Phrase | Search Screen - Advanced Search |  |
|  |  |  | Database - MEDLINE with Full Text |  |
| S5 | (MH "Obstetrical Forceps") | Expanders - Apply equivalent subjects | Interface - EBSCOhost Research Databases | 1,651 |
|  |  | Search modes - Boolean/Phrase | Search Screen - Advanced Search |  |
|  |  |  | Database - MEDLINE with Full Text |  |
| S4 | (MH "Extraction, Obstetrical+") | Expanders - Apply equivalent subjects | Interface - EBSCOhost Research Databases | 3,417 |
|  |  | Search modes - Boolean/Phrase | Search Screen - Advanced Search |  |
|  |  |  | Database - MEDLINE with Full Text |  |
| S3 | S1 OR S2 | Expanders - Apply equivalent subjects | Interface - EBSCOhost Research Databases | 2,781,672 |
|  |  | Search modes - Boolean/Phrase | Search Screen - Advanced Search |  |
|  |  |  | Database - MEDLINE with Full Text |  |
| S2 | (MH "Health Personnel+") | Expanders - Apply equivalent subjects | Interface - EBSCOhost Research Databases | 504,882 |
|  |  | Search modes - Boolean/Phrase | Search Screen - Advanced Search |  |
|  |  |  | Database - MEDLINE with Full Text |  |
| S1 | TI ( midwife or midwives or midwifery or nurse-midwife or obstetrician or doctor or physician or dr or ob or obstetrics or or nurse-midwives or obstetric nurse or nurse or trainee or registrar or practitioner or personnel or resident or medical officer or medical or provider or worker or specialist or attendant or graduate or professional or inter ) OR AB ( midwife or midwives or midwifery or nurse-midwife or obstetrician or doctor or physician or dr or ob or obstetrics or or nurse-midwives or obstetric nurse or nurse or trainee or registrar or practitioner or personnel or resident or medical officer or medical or provider or worker or specialist or attendant or graduate or professional or inter ) | Expanders - Apply equivalent subjects | Interface - EBSCOhost Research Databases | 2,558,224 |
|  |  | Search modes - Boolean/Phrase | Search Screen - Advanced Search |  |
|  |  |  | Database - MEDLINE with Full Text |  |
